# Genome wide association study to identify predictors for severe skin toxicity in colorectal cancer patients treated with cetuximab

**DOI:** 10.1371/journal.pone.0208080

**Published:** 2018-12-17

**Authors:** Jara Baas, Lisanne Krens, Stefan Bohringer, Linda Mol, Cornelis Punt, Henk-Jan Guchelaar, Hans Gelderblom

**Affiliations:** 1 Department of Clinical Oncology, Leiden University Medical Center, Leiden, The Netherlands; 2 Department of Clinical Pharmacy and Toxicology, Leiden University Medical Center, Leiden, The Netherlands; 3 Department of Medical Statistics, Leiden University Medical Center, Leiden, The Netherlands; 4 Clinical Trial Department, Comprehensive cancer center The Netherlands, Nijmegen, The Netherlands; 5 Department of Medical Oncology, Academic Medical Center Amsterdam, Amsterdam, The Netherlands; Lawrence Berkeley National Laboratory, University of California, Berkeley, UNITED STATES

## Abstract

EGFR-antibodies are associated with significant skin toxicity, including acneiform rash and folliculitis. It remains impossible to predict the occurrence of severe skin toxicity due to the lack of predictive markers. Here, we present the first genome-wide association study (GWAS) to find single nucleotide polymorphisms (SNPs) associated with EGFR inhibitor-induced skin toxicity using data of the multicentre randomized phase III CAIRO2 trial (clinicaltrials.gov NCT00208546). In this study, advanced or metastatic colorectal cancer patients were treated with capecitabine, oxaliplatin and bevacizumab with or without cetuximab. Germline DNA was available in 282 of the 368 patients in the cetuximab arm. Mild skin toxicity occurred in 195 patients (i.e. CTC grade 1 or 2, respectively 91 and 104 patients) and severe skin toxicity (i.e. grade 3) in 36 patients. Grade 4 skin toxicity did not occur. None of the SNPs reached the formal genome wide threshold for significance of 5x10^-8^, though SNPs of at least 8 loci did show moderate association (p-value between 5x10^-7^ and 5x10^-5^) with the occurrence of grade 3 (severe) skin toxicity. These SNPs did not overlap with SNPs associated with cetuximab efficacy as found in a previous GWAS in the same CAIRO2 cohort. If formally proven by replication, the SNPs associated with severe EGFR induced skin toxicity may be helpful to predict the occurrence and severity of skin toxicity in patients that will receive cetuximab and allow for adequate information on the risk of skin toxicity and prophylactic measurements.

## Introduction

Monoclonal antibodies directed against the epidermal growth factor receptor (EGFR) are proven to be active anti-tumor agents in colorectal cancer (CRC) and squamous cell carcinoma of the head and neck (SCCHN), either alone or combined with chemo- and/or radiotherapy. These antibodies, cetuximab and panitumumab, are associated with few (non- haematological) adverse events, except for skin toxicity (acneiform rash and folliculitis) which may be significant[1, 2]. Various studies have aimed to find predictive markers to select patients at risk for developing (severe) skin toxicity, as previously reviewed[3]. Among these are studies exploring the predictive value of germline polymorphisms and mutations[4–7]. None of these markers are used in daily practice. However, these studies are hampered by the fact that until now only candidate gene based approaches have been applied. This approach has important limitations since the mechanism of EGFR inhibitor-induced skin toxicity is not completely understood and candidate gene based studies focus only on mutations and genes assumed to be involved by biological plausibility, such as EGFR polymorphisms[4] and EGFR gene copy number variants[7]. A genome-wide approach allows identifying new and yet unknown single nucleotide polymorphisms (SNPs) possibly leading to new insights on the pathophysiology of EGFR inhibitor-induced skin toxicity. Indeed, GWAS approaches have shown to be successful especially in finding genetic biomarkers for drug toxicity[8]. Establishing advance identification of patients who are at risk of developing (severe) skin toxicity is of value because it allows better information to individual patients on the expected (extent) of side effects. Moreover, in case of an expected high risk for severe skin toxicity one might choose to start prophylactic therapy such as a tetracycline derivative[9], since it is well known that EGFR inhibitor-induced skin toxicity negatively influences quality of life[10]. Here we present the first GWAS to identify SNPs associated with EGFR inhibitor-induced skin toxicity using data of the multicenter randomized phase III CAIRO2 trial of the Dutch Colorectal Cancer Group (DCCG)[11]. The CAIRO2 trial is registered at clinicaltrials.gov (NCT00208546).

## Methods

### Patients

Germline DNA was obtained after written informed consent from patients participating in the CAIRO2 trial and randomized to receive capecitabine, oxaliplatin and bevacizumab (CAPOX-B) with or without cetuximab. The study was approved by the Committee on Research involving Human Subjects Arnhem-Nijmegen (the Netherlands) and by all local institutional ethics boards. All patients gave written informed consent. Cetuximab was administered intravenously with an initial dose of 400mg/m^2^ followed by 250mg/m^2^ weekly, until progression of disease, unacceptable toxicity or death, whichever occurred first. Inclusion criteria of the CAIRO2 trial are described in detail elsewhere[11]. Briefly, the study included previously untreated patients with locally advanced or metastasized colorectal cancer. Skin toxicity was graded according to the National Cancer Institute Common Toxicity Criteria (NCI-CTC) version 3.0, and cetuximab-associated skin toxicity was defined as any adverse cutaneous event other than hand-foot syndrome. Cetuximab-associated skin toxicity reported during the first nine weeks of the study was included in the present analysis. This was chosen to do so to minimize the influence of antitumor efficacy on the results, since responders have longer exposure to cetuximab and therefore have more chance developing toxicity (the first CT response evaluation was done at 9 weeks). Moreover, skin toxicity typically starts within the first 1–2 weeks after start of cetuximab [12], further supporting the inclusion of data obtained during only the first 9 weeks of treatment.

For each patient, the worst grade of skin toxicity reported during the first nine weeks on study was used in the current analysis. It was chosen to divide the study population into two distinct phenotypes; patients with grade 3 (or higher) toxicity and patients with no or up to grade 2 skin toxicity, based on the clinical relevance of grade 3 versus grade 1 and 2.

All analyses were corrected for gender and age effects. Information on race was not available from the CAIRO2 database. The CAIRO2 protocol did not describe how to prevent or manage skin toxicity, as the available evidence of (pre-emptive) therapy was limited at that time. Therefore, it was not feasible to correct for prescribed medication (for skin toxicity). Since this GWAS cohort was previously studied to find biomarkers for cetuximab efficacy [13], it was possible to compare whether these cetuximab efficacy markers and the skin toxicity biomarkers found in the present study were overlapping. Markers associated with toxicity but not efficacy were considered to be most valuable, as these might identify patients at risk for toxicity without modifying likelihood of efficacy.

### Genotyping

Whole blood was collected at baseline and germline DNA was isolated from peripheral leukocytes using MagnaPure Compact (Roche diagnostics, Almere, the Netherlands). Genotyping was performed on Human OmniExpress v12 BeadChip arrays containing 733,202 markers (Illumina, San Diego, CA, USA). Genotype calls were set using GenomeStudio software (Illumina). The following cut-off values were used to filter out incorrectly called genotypes: GenCall ≥ 0.85; ClusterSep ≥ 0.3; CallFreq >0.85; AB T-mean 0.2–0.8, resulting in the exclusion of 3172 markers (0.43%).

### Statistical analysis

In total, 584,109 markers entered the quality control. Plink version 1.07 was used for the quality control. Allele frequencies were filtered at 5%, excluding 3,071 markers (0.5%). No markers failed further QC based on missingness of genotypes (cut-off set at 3%). Hardy-Weinberg equilibrium (HWE) was evaluated per remaining marker using a χ^2^ goodness-of-fit statistic. Markers were excluded based on a P-value with a cut-off P-value ≤ 1.0^−7^. One marker failed this test (0%). Based on these quality controls 581,037 markers remained in the analysis.

Quality control of persons was performed by missing data analysis (threshold at 2%) and gender check. None of the individuals failed the quality control. Multi-dimensional scaling (MDS) was performed in order to detect possible population stratification (MDS plot is added as a supplemental graph). The sample was judged not to contain distinct clusters or outliers, such that no individuals were excluded. Also MDS-coordinates had not to be used to correct for population stratification. MDS analysis from plink was used for analysing population stratification. After quality controls SNPs were imputed using software *impute2* without pre-phasing using the `The Genome of the Netherlands`(GoNL) panel, which is the freely available panel that most closely matches the study population (www.nlgenome.nl). In total, data was imputed to 5.830.976 SNPs.

We have analyzed genetic association using binary logistic regression, correcting for age and sex. The outcome was coded as skin toxicity grade greater or equal to three against smaller than three. We also evaluated a model contrasting skin toxicity grade greater or equal to two versus smaller than two and an ordinal logistic regression for which skin toxicity was treated as an ordinal outcome. For the latter two models no P-value was smaller than 10^-6 and results are not shown.

Locations of SNPs were identified using data from the Hapmap project (www.hapmap.org) and RegulomeDB (http://www.regulomedb.org/), expression of the genes harbouring the SNPs was determined using GTEx (https://www.gtexportal.org/home/) and functions of genes were determined using the NCBI Gene database (http://www.ncbi.nlm.nih.gov/gene).

The Cut-off value significance was set at 5x10^-8^ in this GWAS.

## Results

### Patient characteristics

Germline DNA was available from 282 of 368 patients receiving cetuximab. Baseline characteristics of the patients are listed in Table 1 and are similar to the baseline characteristics of the complete study population included in the CAIRO 2 study. Median progression free survival was 9.4 months (95% CI 8.4–10.5 months). Skin toxicity occurred in 231 patients (82%); 195 (69%) had mild skin toxicity (i.e. grade 1 or 2, respectively 91 and 104 patients) and 36 (13%) had severe (i.e. grade 3) skin toxicity. Grade 4 skin toxicity did not occur. Various types of medication used to treat EGFR inhibitor-induced skin toxicity were used, including antibiotics, steroids and moisturizers. Both systemic and topical antibiotics were tetracyclines (minocycline, doxycycline, tetracycline), macrolides (erythromycin, clarithromycin, clindamycin) and metronidazole. It was not documented whether these agents were used pre-emptively or reactively.

**Table 1:** Baseline characteristics.

	Overall	Skin toxicity grade 0–2	Skin toxicity grade 3	p-value
**Age–years**				0.926*
Median	63	64	61	
Range	33–80	33–80	41–79	
**Gender–n (%)**				0.5355**
Male	183 (65)	157 (64)	26 (72)	
Female	99 (35)	89 (36)	10 (28)	
**WHO performance score–n (%)**				
0	185 (66)	165 (67)	20 (56)	
I	94 (33)	80 (32)	14 (39)	
II	1 (<1)	1 (<1)	-	
Not reported	2 (<1)	-	2 (5)	

* One-way ANOVA

** Pearson Chi-square test

### Genome-wide association (GWA) analysis

**Fig 1:**
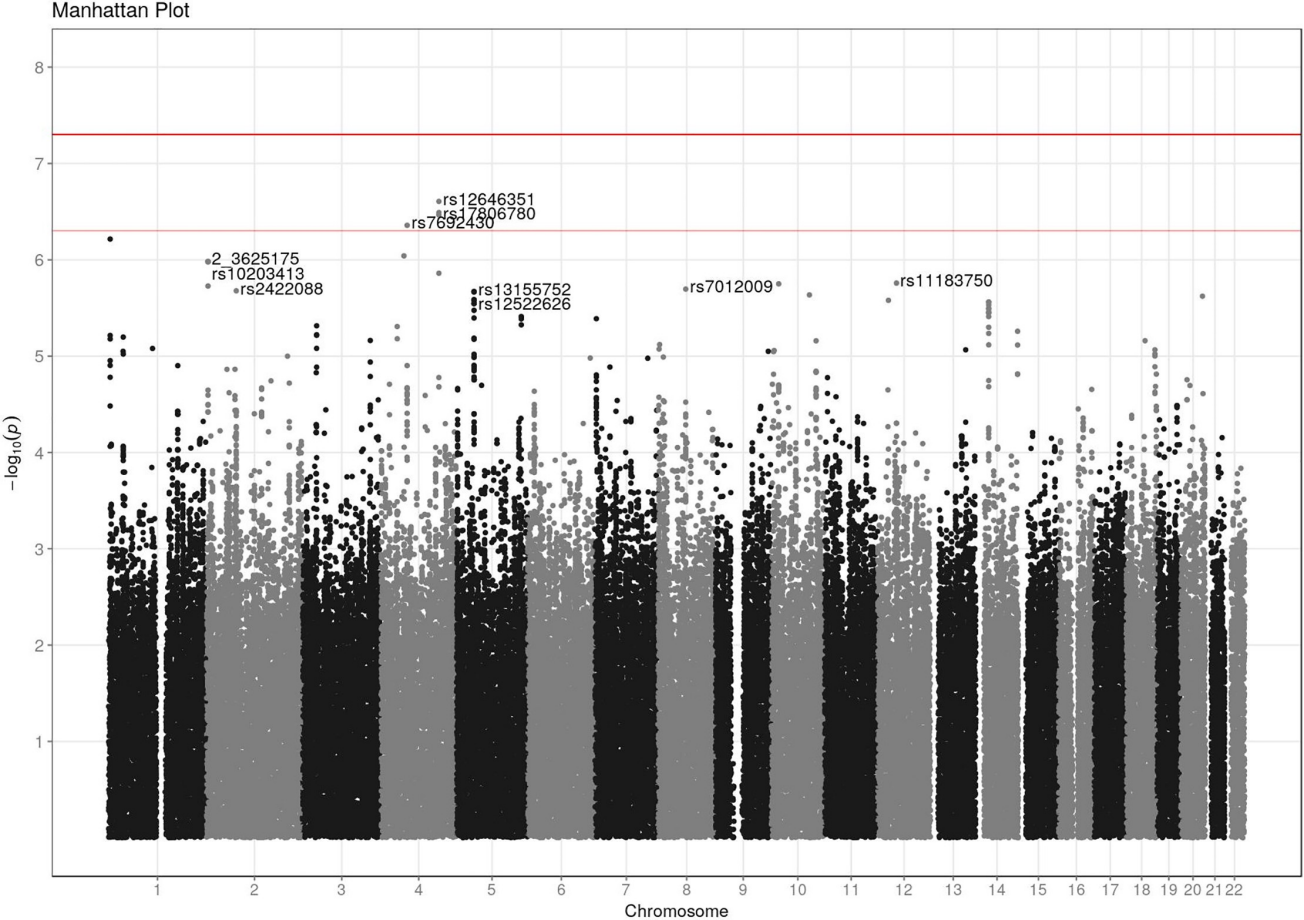
Manhattan plot.

The Manhattan plot of the GWA analysis is shown in Fig 1. None of the SNPs reached the formal threshold for genome wide significance. The inflation factor for this analysis was λ = 1.03, suggesting that P-values were unbiased and confounding such as population stratification were not a problem. Imputation using GoNL did not imply any new loci although the most associated SNP was imputed. Table 2 shows the 10 SNPs with the lowest p-value. Two SNPs per locus are reported; the one with the lowest p-value and the most associated SNP that had not been imputed (reported as R^2^ = 1)–or one SNP in case an un-imputed SNP showed the strongest association. Odds ratio was below 1 for the SNP with the highest p-value indicating a protective effect of the minor allele. Four SNPs were located in a gene. To our knowledge none of these genes have previously been reported to be related to EGFR inhibitor induced skin toxicity.

**Table 2:** Moderate associated SNPs.

Marker	Chromosome	Position	Allele frequency	R^2^	Gene	OR	95% CI	p-value
rs12646351	4	146740625	0.20	0.95	ZNF827	0.04	0.01–0.33	2.47x10^-7^
rs17806780	4	146952104	0.20	1.00	ZNF827	0.04	0.01–0.35	3.42x10^-7^
rs7692430	4	66220697	0.19	0.97	EPHA5	4.57	2.46–8.49	4.39x10^-7^
2_3625175	2	3625175	0.25	0.96	-	0.14	0.05–0.39	1.04x10^-6^
rs10203413	2	3607053	0.25	1.00	RPS7	0.14	0.05–0.39	1.05x10^-6^
rs11183750	12	45803962	0.24	1.00	PCED1B	0.14	0.05–0.40	1.74x10^-6^
rs7012009	8	70753873	0.18	0.94	-	0.04	0.01–0.37	2.01x10^-6^
rs2422088	2	75251361	0.31	0.92	-	0.17	2.44–13.3	2.10x10^-6^
rs13155752	5	44680687	0.40	0.99	-	3.82	2.11–6.92	2.13x10^-6^
rs12522626	5	44685698	0.40	1.00	-	3.82	2.11–6.91	2.26x10^-6^

R2 corresponds to the 'info' column computed by impute2 which can be interpreted as the squared correlation between true and imputed genotypes. Abbreviations: OR, odds ratio; CI, confidence interval; ZNF827, zinc finger protein 827; EPH5, EPH receptor A5; RPS7, 40S ribosomal protein S7; PCED1B, PC-esterase domain-containing protein 1.

## Discussion

To the best of our knowledge this is the first GWAS on predictors for EGFR inhibitor induced skin toxicity. Though none of the SNPs reached the formal threshold of genome wide significance, all reported SNPs were moderately associated (i.e. p-value between 5x10^-7^ and 5x10^-5^)[14, 15]. All of these SNPs showed relatively large effect sizes, owing to the fact that our study was powered to detect only larger effect sizes. Such bigger effects have also reported in other toxicity studies, lending plausibility to our findings.

A complicating aspect is that it is well known that the occurrence of skin toxicity is related to EGFR inhibitor efficacy[16, 17]. However, since this relation is a statistical association rather than a lab-confirmed causal mechanism one might expect that genetic markers associated with skin toxicity and EGFR efficacy are not (fully) overlapping. Indeed, not all patients that experience EGFR induced skin toxicity are responsive to EGFR inhibitor therapy and also not all responsive patients experience skin toxicity[17]. This cohort was previously used to study predictors for efficacy of cetuximab treatment [13], we had the unique possibility to see whether the identified SNPs were also associated with efficacy. None of the 10 SNPs with the lowest p-values showed overlap with the GWA analysis on predictors for efficacy. Therefore the SNPs reported in this study seem to be exclusively associated with toxicity and may be useful for predicting the occurrence of severe skin toxicity in individual patients receiving cetuximab, independently of anti-tumor efficacy.

The odds ratios of 6 of the 10 SNPs with the lowest p-values were below 1, indicating a protective effect of the minor allele. One of these SNPs (rs10203413) is located on a gene encoding for 40S ribosomal protein S7 (RPS7). This protein has not been linked to EGFR-inhibiting agents before. However, mitochondrial RPS7 is overexpressed in dermal papilla cells with high tendency of aggregation, which produce growth factors stimulating proliferation of follicular epithelium[18]. EGFR inhibitor-induced skin toxicity is typically most distinct in the seborrhoeic areas of the skin. We hypothesize that this SNP leads to decreased activity of RPS7 and thereby decreased follicular proliferation, thereby leading to lower susceptibility to EGFR inhibitor-induced skin toxicity.

One group of SNPs is located on a gene encoding zinc finger protein 827 (ZNF827; rs17806780). The function of this gene is yet unknown and it has not been related to EGFR-inhibiting agents or dermal cells before and expression in skin tissue is average compared to other organs. Zinc finger proteins are small protein domains characterized by the presence of one or more zinc ion to stabilize their structure. There is a broad variance in structure of these proteins and they contribute to a variety of cellular processes, including cell signalling, proliferation and apoptosis. This gene is close to (among others) SMAD1, a gene active in the bone morphogenic protein-7-phosphorylated Smad1, -5, -8 (BMP7-p-Smad1/5/8) pathyway. This pathway is mainly known for its role in osteosynthesis however it has been described to be involved in cetuximab resistance in oral squamous cell carcinomas[19]. While there was no overlap with the GWA analysis on predictors for efficacy [13], it could well explain a relation with EGFR inhibitor induced skin toxicity since this type of toxicity is related to anti-tumor efficacy. Another group of SNPs (top SNP rs7692430) is located on EPH receptor A5 (EPHA5). This gene belongs to the ephrin subfamily of the tyrosine kinase receptor family, and is involved in various developmental processes including oncogenesis[20]. It has however never been linked to EGFR-inhibiting agents or any form of skin toxicity before and expression in skin tissue is low. As for neighbouring genes of EPHA5, none of them have been described to be related to follicular proliferation of other processes in skin tissue. Hypothesizing on relation of these genes to EGFR inhibitor-induced skin toxicity remains complicated, since the exact pathophysiology of EGFR inhibitor-induced skin toxicity is still not fully elucidated.

As previously discussed, age, performance status and race might be associated with the occurrence of (severe) skin toxicity, although studies remain inconclusive so far[3]. Patients with grade 0–2 and grade 3 skin toxicity had comparable age and performance score, as shown in Table 1. Information on race was not available from the original CAIRO2 database, however when using the information of multi-dimensional scaling (MDS) analysis it is possible to predict (diversity of) ethnicity of the cohort. Inspection of the plots of MDS-coordinates in combination with a low inflation factor seems to exclude the presence of several different ethnicities in our sample. As this was a Dutch study we presume that almost all patients were Caucasian (though there might me some variation due to the multi-ethnic composition of the population in the Netherlands). Furthermore, it we decided not to correct for medication used for skin toxicity. The original database of the CAIRO2 did not contain information on whether these medications were used preventive or reactive. However, since little was published about effective prevention of EGFR inhibitor-induced skin toxicity at time of the CAIRO2 study and this was not in the guidelines, it is highly unlikely that accurate prevention took place. If our results were confounded by medication we would expect our results to be diluted by individuals with lower skin-toxicity that should have really been reported as grade 3 patients, thereby making our results conservative.

The CAIRO2 study specified to use NCI CTC version 3.0, grading skin toxicity based on the need of therapy (grade 1 versus 2) and the presence of symptoms such as pain, disfigurement or ulceration (grade 3). The cohort was divided into two distinct phenotypes; patients with grade 3 (or higher) toxicity and patients with no or up to grade 2 skin toxicity It was chosen to do so since the occurrence of grade 3 toxicity is of significant clinical relevance; toxicity of this severance might interfere with anti-tumor efficacy due to the need for dose reductions, dose delays and, in some cases, even permanent discontinuation of the EGFR inhibitor. Nowadays, version 4.0 is being used, grading acneiform rash by the percentage of body surface area (BSA) affected, the need for oral of systemic antibiotics and the impact on activities of daily living (ADL). This newer approach lends towards a more strict classification of skin toxicity. Nonetheless, the grading system of version 3.0 was sufficient to divide the current cohort into two distinct phenotypes, identifying a group of patients with clinically significant toxicity. Notably, 19% of this cohort did not experience any skin toxicity at all. However, we have previously demonstrated that up to 24% of patients treated with cetuximab (either alone or in combination with chemotherapy) remain free from skin toxicity[3].

Finding an identical population for replication of these results is hampered by the fact that the CAIRO 2 study showed inferior survival patients receiving the combination of cetuximab and bevacizumab, and this schedule has not been used since. Nonetheless, as it is unlikely that the addition of bevacizumab influences EGFR inhibitor–induced skin toxicity, a cohort treated with a comparable treatment schedule except for not receiving bevacizumab seems appropriate. The choice of the genetic model is another consideration in this (and other) GWA studies. It is conceivable that especially recessive effects, i.e. effects due to absence of two functional alleles, might play a role in side effect studies. For SNPs in linkage disequilibrium (LD) with such causal sites the apparent genetic model would move towards an additive model with decreasing LD [21]. We believe that using dense, imputed genotypes makes it unlikely that we missed important findings. However, we plan to investigate different disease models in future, exploratory work.

While the end goal of studying the association of SNP markers with toxicity is establishing a prediction model, the lack of significant findings in our study precludes that goal. We have avoided the construction of a prediction model in the absence of significant findings to avoid over-optimistic interpretation. The putative associations reported here might still be used in meta-analyses, guide research for associations in new studies, and might suggest biological functions though database searches. In conclusion, at least 8 loci were shown to be moderately associated with EGFR inhibitor induced severe skin toxicity and none were associated with anti-tumor efficacy. For most of these loci we were able to find a plausible biological rationale for their relationship with skin toxicity of EGFR inhibition. The effect sizes were relatively high. Though routine testing for those SNPs might not be part of routine practice in the near future, if replicated, these results will lead to new insight and future research on the most optimal risk stratification strategy.

## Supporting information

S1 Fig Scatterplot of the first two genetic principal components. (TIF)
