# Supplementary figures and images for: Genome wide association study to identify predictors for severe skin toxicity in colorectal cancer patients treated with cetuximab
Source: PLoS One. 2018 Dec 17;13(12):e0208080. doi: 10.1371/journal.pone.0208080 (PMC6296548; doi:10.1371/journal.pone.0208080)

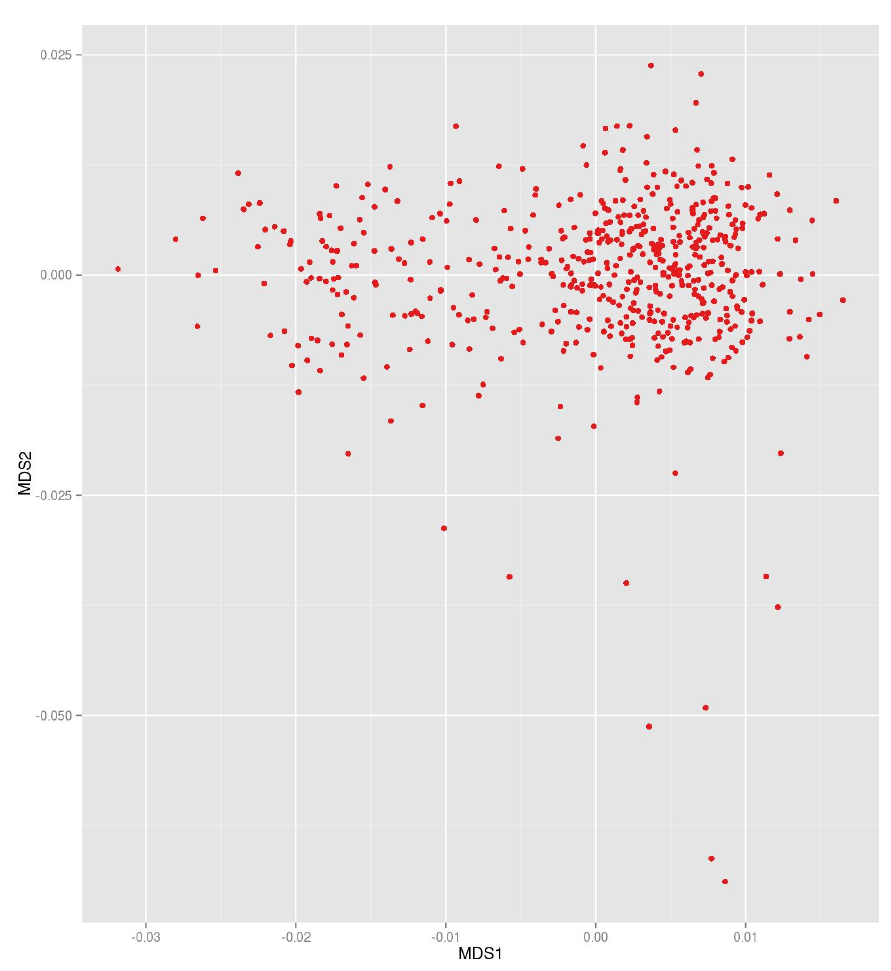

Supplement: S1 Fig — (TIF) [file pone.0208080.s001.tif]
